# Vascular type Ehlers-Danlos syndrome is associated with platelet dysfunction and low vitamin D serum concentration

**DOI:** 10.1186/s13023-016-0491-2

**Published:** 2016-08-03

**Authors:** Albert Busch, Sabine Hoffjan, Frauke Bergmann, Birgit Hartung, Helena Jung, Daniela Hanel, Andeas Tzschach, Janos Kadar, Yskert von Kodolitsch, Christoph-Thomas Germer, Heiner Trobisch, Erwin Strasser, René Wildenauer

**Affiliations:** 1 Department for General, Visceral, Vascular & Paediatric Surgery, University Hospital of Würzburg – ZOM, Oberduerrbacher str 6, 97080 Würzburg, Germany; 2 Department of Human Genetics, Ruhr-University, Bochum, Germany; 3 MVZ wagnerstibbe, amedes Gruppe, Hannover, Germany; 4 Institute of Clinical Chemistry, University Hospital Würzburg, Würzburg, Germany; 5 Institute for medical genetics and molecular medicine, Cologne, Germany; 6 synlab MVZ Stuttgart GmbH, Stuttgart, Germany; 7 Institute of Clinical Genetics, Technische Universität Dresden, Dresden, Germany; 8 UniversitätsCentrum für Seltene Erkrankungen, University Hospital Carl Gustav Carus, Dresden, Germany; 9 Laboratory for Transfusion Medicine, Cologne, Germany; 10 Department of Cardiology, University Heart Center, University Hospital Hamburg-Eppendorf, Hamburg, Germany; 11 Laboratory and Ambulance for Coagulation Disorders, Duisburg, Germany; 12 Transfusion Medicine and Haemostaseology Department, University Hospital Erlangen, Erlangen, Germany

**Keywords:** Vascular type Ehlers-Danlos syndrome, EDS, Bleeding disorder, Platelet dysfunction, Vitamin D

## Abstract

**Background:**

The vascular type represents a very rare, yet the clinically most fatal entity of Ehlers-Danlos syndrome (EDS). Patients are often admitted due to arterial bleedings and the friable tissue and the altered coagulation contribute to the challenge in treatment strategies. Until now there is little information about clotting characteristics that might influence hemostasis decisively and eventually worsen emergency situations.

**Results:**

22 vascular type EDS patients were studied for hemoglobin, platelet volume and count, Quick and activated partial thromboplastin time, fibrinogen, factor XIII, von Willebrand disease, vitamin D and platelet aggregation by modern standard laboratory methods. Results show a high prevalence of over 50 % for platelet aggregation disorders in vascular type EDS patients, especially for collagen and epinephrine induced tests, whereas the plasmatic cascade did not show any alterations. Additionally, more than half of the tested subjects showed low vitamin D serum levels, which might additionally affect vascular wall integrity.

**Conclusion:**

The presented data underline the importance of detailed laboratory screening methods in vascular type EDS patients in order to allow for targeted application of platelet-interacting substances that might be of decisive benefit in the emergency setting.

**Electronic supplementary material:**

The online version of this article (doi:10.1186/s13023-016-0491-2) contains supplementary material, which is available to authorized users.

## Background

Ehlers-Danlos syndrome (EDS) is a rare connective tissue disorder with multitude heterogeneous symptoms. The estimated overall incidence is approx. 1:5000 [1]. Mutations of fibrillar collagens (I, II, III, IV, V, VI, IX and XII) or enzymes involved in their biosynthesis (i.e. *PLOD1*, *CHST14*) are responsible for the multi-systemic affection involving skin, ligaments, muscles, vessels, hollow organs, eyes and teeth [2, 3]. The rarity, although good in itself, impedes both, evidence or eminence-guided treatment, especially in emergency situations.

Classifications of EDS based on symptoms in the Berlin Nosology from 1986 aimed to facilitate diagnosis and genetic counseling and to provide treatment suggestions. In the currently applicable Villefranche Classification from 1997 these were additionally linked to the respective genetic mutations [3, 4].

Vascular type EDS, formerly known as EDS type IV, comprises approximately 8 % of all EDS patients [5]. Mutations in *COL3A1* cause stigmata like thin translucent skin with visible venous pattern, congenital clubfoot or hip dislocation and characteristic facial appearance. However, vascular fragility and abnormal vessel structure, cause the characteristic symptoms like easy bruising, severe varicosities, arterial dissection or aneurysm formation and eventual arterial or intestinal rupture, that can lead to fatal consequences and a reduced life span of patients [2, 5, 6].

Current treatment is symptomatic only, since no causal therapy exists. The gold standard “conservative first” is, however, of limited advice, for most admissions are due to emergency bleedings, involving mid-size and big arteries in thoracic or abdominal position, including the aorta [7, 8]. Accordingly, mortality rates after emergency surgery are high and have only modestly declined from 40–60 % to around 30–40 % with evolving endovascular arterial repair [8–10].

Moreover, secondary intervention rates up to 50 % due to access site, as well as unrelated secondary bleeding complications add to an unacceptable morbidity and mortality rate in this subset of patients [8, 9, 11, 12]. The critical points impairing surgical outcome are vascular fragility, probably worsened by circulating collagenases during acute events of bleeding, and a deficient coagulation cascade impairing primary healing [13, 14]. Therefor, desmopressin administration prior to surgery was recommended in EDS patients [15]. But whether defects of pro-coagulant factors or misleading platelet-collagen-interactions are causative for deficient coagulatory properties is still unclear and little is known about the coagulation cascade in vascular type EDS in particular [16].

We therefore aimed to investigate hemostasis in vascular type EDS patients under normal conditions by standard laboratory means in order to reveal coagulation defects, eventually allowing a targeted therapy to breach the fatal course of acute bleedings in this subset of patients.

## Methods

### Patient identification and sample analysis

Vascular type EDS patients were identified and invited with support of a nationwide patients’ self-help group, the German National EDS Initiative, in accordance with the national regulations for protection of data privacy. Inclusion criterion was affection by vascular type EDS. All participants were in good condition (Karnofsky Index >90 %). The study was in accordance with the declaration of Helsinki and approved by the local ethics committee (University Hospital of Würzburg) and with patients’ informed and written consent. The study was conducted between January 1st and December 31st 2015.

Blood was drawn from a cubital vein with free flow after initial stasis. In order to allow immediate analysis of the samples, seven different laboratories (affiliations no. 3, 4, 5, 6, 9, 10, 11) across Germany participated in the study. All tests are standardized, commercially available products with defined and validated normal ranges and criteria for pathologic results. Normal measurement ranges and availability of single test methods varied slightly among the participating laboratories (Additional file 1: Table S1 and Additional file 2: Table S2). All laboratories are subject to routine quality control and daily/weekly calibration of their test series.

### Basic test methods

Automated analyzers did hemograms, which delivered hemoglobin (Hb), platelet count and optional thrombocyte volume. C-reactive protein (CRP) serum concentration was measured as a reference value to interpret test results and exclude acute inflammatory processes. Prothrombin time according to Quick and activated partial thromboplastin time (PTT) for the extrinsic and intrinsic coagulation pathway were measured by a turbidimetric assay. Depending on the laboratory Fibrinogen was measured either by the Clauss-method or as prothrombin time derived fibrinogen (all Quick values were within normal range, Additional file 1: Table S1).

### Factor XIII

Factor XIII (FXIII) was measured either by a chromogenic assay or ELISA (enzyme linked immunosorbent assay) since it is not detected elsewhere, yet has a distinctive function in fibrin crosslinking.

### Von Willebrand diagnostics

Von Willebrand factor (vWF) was assessed by two different assays. Latex Immunoassay or ELISA-methods were applied to measure vWF-antigen (vWF:ag) and vWF-activity (vWF:act). Reduced vWF (low vWF:ag) and reduced activity (low vWF:act) in synopsis with a clinical bleeding phenotype was thus defined as possible von Willebrand syndrome (vWS). The patients’ blood group was determined in order to allow correct assess correct normal ranges.

### Vitamin D

Vitamin D concentration was measured from plasma samples by high performance liquid chromatography (HPLC) or enzyme immunoassay (EIA).

### ROTEM®

The ROTEM® is a haemostasis analyzer, that measures kinetic changes of the clot elasticity of whole blood samples [17]. It is provided by the TEM group (Basel, Switzerland) (http://www.rotem.de/en) and allows quantitative and qualitative assessment by measuring different parameters of the clot status of the blood sample. In our study we applied the in-tem® (fast assessment of clot formation, fibrin polymerization and fibrinolysis via the intrinsic pathway), ex-tem® (fast assessment of clot formation, fibrin polymerization and fibrinolysis via the extrinsic pathway), fib-tem® (fast analysis without platelets; qualitative assessment of fibrinogen status) and ap-tem® (fast detection of lysis when compared with ex-tem via fibrinolysis inhibition).

### PFA100®

The PFA-100® analyzes platelet function from citrate blood by aspiration at high shear rates through disposable cartridges containing an aperture within a membrane coated with collagen (Col) plus either epinephrine (EPI) or ADP (ADP). These agonists induce platelet adhesion, activation and aggregation and lead to occlusion of the membrane and cessation of the blood under normal conditions. The resulting time until cessation is called closure time (CT) (http://www.practical-haemostasis.com/Platelets). CT-Prolongation is thus suggestive of deranged primary hemostasis such as thrombocyte dysfunction by impaired activation and cross-linking or vWS. Requirements are a thrombocyte count >80x10^6^/mm^2^ and a hematocrit >35 %.

### Light transmission aggregometry by Born

Born aggregometry is based on the principle of light transmission aggregometry. Platelet rich plasma is stirred in a cuvette and different agonists are added to induce aggregation. Aggregated platelets absorb less light and transmission detected by a photocell thus increases (http://www.practical-haemostasis.com). Agonists are collagen (Col), epinephrine (EPI), adenosintriphosphate (ADP) and ristocetin (Rist). Additionally, thrombin, thromboxaneA2 and different agonist concentrations can be used. This multiple testing allows measurement of aggregation, shape change of platelets whilst activation and degranulation.

Values are normally displayed in percentage of transmitted light compared to a control probe (blank) with platelet poor plasma set to 100 % light transmission. Results below the normal range thus indicate disturbed aggregation of platelets.

## Results

### Vascular type EDS patients have heterogeneous symptoms and genetics

22 individuals with the diagnosis of vascular type EDS were enrolled in the study, including fourteen females and eight males (median age 37 ± 16 years). Among them ten patients descended from three families (family A: ID5 and 6; family B: ID16-19; family C: ID11-14) and another three participants reported EDS symptoms in first-degree relatives not enrolled in the study (Table 1).

**Table 1:** Characteristics of the study population: method of diagnosis shows either the mutation in *COL3A1* if found, diagnosis by EDS specific electron microscopy of antebrachial skin biopsy or clinical phenotype evaluated by a geneticist. Major and minor symptoms include EDS and especially vascular type EDS specific symptoms based patients’ reports. Familial involvement shows positive family history (yes) in patients and A, B, C refer to distinct families, where more than one member participated in the study (Fig. 2). Medication shows daily and occasional drugs at the time of investigation. (y = years, f = female, m = male, SAB = subarachnoideal bleeding)

ID	Sex	Age	Method of EDS diagnosis	Major symptoms	Minor symptoms	Family involvement	Medication
		(y)					
1	f	39	*clinical*	-	(uncomplicated delivery)	yes	Celiprolol
2	f	45	c3508G > A (p.G1170S)	hemorrhage	(mother/sister had lethal internal bleedings)	yes	-
3	f	44	Col3A1 c.1804C > A	hemorrhage, hypermenorrhea	(sister/niece have same vasc EDS mutation)	yes	Thyroxine, Venlafaxine
4	m	52	skin biopsy	cranial aneurysm, SAB	-	-	ASS, Metoprolol, Amiodarone
5	m	43	COL3A1:c.{973G > T};{=}	cranial aneurysm	-	A	-
6	m	13	COL3A1:c.{973G > T};{=}	-	hypermobility	A	-
7	f	22	c.2295_2312delTCCTATTGGTCCTCCTGG,p,lle767_Pro772del	vertebral aneurysm	hypermenorrhea	-	evtl Tranexamic acid
8	f	36	*clinical*	-	hypermobility	-	Thyroxine
9	m	53	*clinical*	-	multiple hernia	-	-
10	f	25	skin biopsy	internal bleeding	hypermobility,kissing spleen,varicose veins,valve insuffic	-	Tramadol,Bisoprolol,Duloxetine
11	f	75	COL3A1:IVS16 + 1G > A	cervical av-istula	hemorrhage, varicose veins, hypermobility	C	-
12	f	46	COL3A1:IVS16 + 1G > A	kidney infarction, SAB	hemorrhage, hematoma lower extremity, hypermobility	C	-
13	f	55	COL3A1:IVS16 + 1G > A	SAB	hypermobilita	C	-
14	f	29	COL3A1:IVS16 + 1G > A	-	spina bifida, hydrocephalus, hypermobility	C	-
15	f	41	*clinical*	atrial septum aneurysm	hemorrhage, hypermobility, mitral valve reflux	-	Nebivolol,Kodein,Prednisolon
16	f	50	*skin biopsy, negative* *Col5A/Col3A testing*	hemorrhage	(six uncomplicated deliveries)	B	-
17	m	19	*clinical*	gastric perforation	hemorrhage	B	-
18	m	8	*clinical*	hemorrhage	impaired wound healing	B	-
19	m	9	*clinical*	-	facial stigmata of EDS	B	-
20	m	50	Col3A1 c.3544G > A	retroperitoneal hemotoma	-	-	-
21	f	25	Col3A1c.1258G > C	carotid dissection, stroke	contralateral asymptomatic dissection	-	-
22	f	49	skin biopsy	hemorrhage	gestational bleeding	-	-

Major and minor symptoms varied tremendously, with abnormal bleedings, aneurysm formation, unusual hemorrhage and also non-vascular EDS symptoms. Eleven participants had defined mutations in *COL3A1*, three were diagnosed with an EDS specific skin biopsy by electron microscopic examination and eight were diagnosed by clinical evaluation of a geneticist (Table 1).

### Blood count and plasmatic coagulation are not altered in vascular type EDS

Hemograms showed no alterations for Hb (13.6 ± 1.1 mg/dL; different normal ranges for males and females were respected – data not shown) and platelet count (276 ± 55.4×10^6^/mm^2^). Platelet volume (9.9 ± 0.4 fL) was available from eleven individuals, and only one patient (ID8) showed an aberrant volume of 5.8 fL. CRP was under the threshold of 0.5 mg/dL in all patients (Additional file 1: Table S1).

Prothrombin time, PTT, Fibrinogen and FXIII-levels represented plasmatic coagulation. Measurements were performed in almost every patient. Quick (97.4 ± 14.3 %) and PTT (30.6 ± 3.4 s) showed normal results for almost all participants. Fibrinogen (2.8 ± 0.6 g/L) and FXIII (114.7 ± 23.5 μg/dL) showed borderline levels for only one patient each, but were within normal ranges otherwise (Additional file 1: Table S1). Additional ROTEM® analysis was performed to study clot formation and fibrinolysis within the first subjects enrolled in the study – was, however, abandoned due to high costs or un-availability (data not shown).

Von Willebrand factor diagnostics were available for thirteen patients (Additional file 3: Figure S1/Additional file 2: Table S2). One patient (ID8) showed reduced values and along with a clinical bleeding diathesis was assumed to suffer from a mild form of vWS. Patient ID3 showed abnormally high values and was supposed to eventually suffer from a collagen-receptor associated platelet disorder (assumption by the respective laboratory consultant). The laboratory diagnosis of subnormal vWF-values was thus made in only 1 out of 13 patients.

### Platelet function is impaired in vascular type EDS

All enrolled individuals were screened for thrombocyte function by either PFA100® or light transmission aggregometry according to Born, depending on availability. For eight patients both were available.

**Fig. 1:**
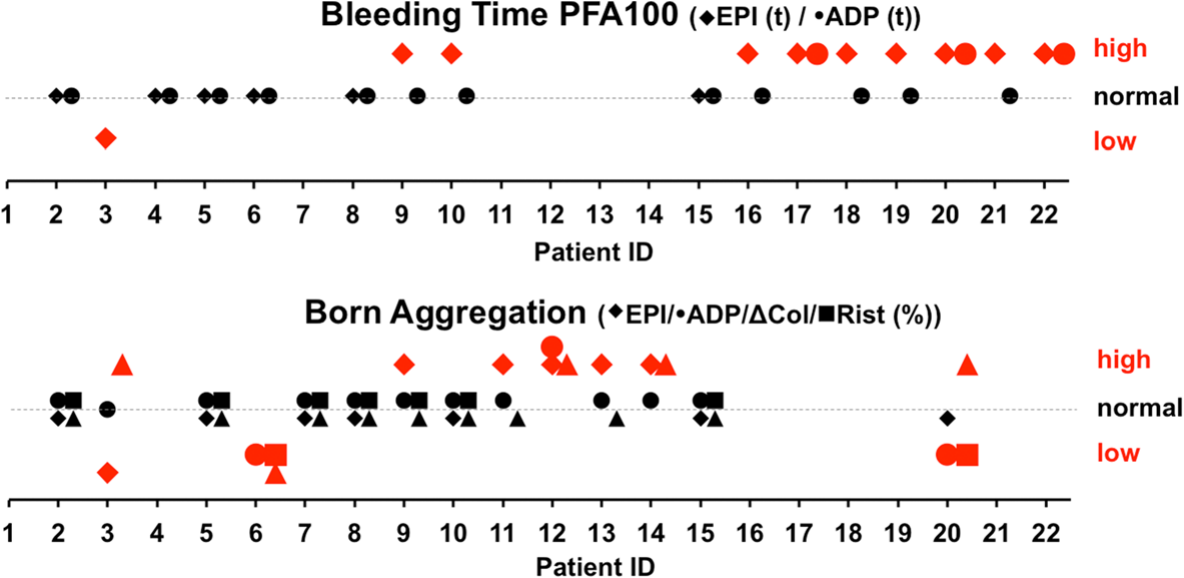
Platelet function diagnosis: The graphs show qualitative alterations in platelet function diagnosis by PFA100® (upper) or light transmission aggregometry (lower). Bold red signs demonstrate deviation from the normal range (dotted line). Bleeding time is reported in seconds (s), higher values thus indicating an abnormal long time till aggregation. Born Aggregation is reported in percentage of a normal probe with no aggregation and complete light transmission, higher values thus indicating impaired aggregation. Abbreviations in brackets indicate stimulating substances for test: EPI = epinephrine, ADP = adenosintriphosphate, Col = collagen; Rist = ristocetin; The quantitative values for each patient and test are depicted in Additional file 2: Table S2

In total,  eleven of 22 patients (50 %) showed impaired aggregation, especially after induction with epinephrine (EPI) and collagen (Col) in at least one of two methods (Fig. 1). Additional four patients showed abnormal results of unclear significance. Six patients showed aberrant values in ADP interaction. From eight patients, in whom both test methods were available, six showed coherent results.

### Vascular symptoms and thrombocyte dysfunction vary within families

Three families with more than one member were enrolled. Families A and C had mutations in *COL3A1*, identically found in all affected members (Table 1). The index person in family B was diagnosed by clinical phenotype by a geneticist, as screening for *COL3A1/COL5A1* mutations and analysis of skin biopsy did not show clear results.

Symptoms or alterations in coagulation parameters varied substantially, even within genetically uniform families. In family A the phenotype of EDS by facial appearance was much more pronounced in the son than the father along with clear signs of platelet dysfunction in the offspring (Fig. 1). In contrast, members of family B were more similar in phenotype and symptoms and showed similar alterations in PFA100® measurements (Additional file 2: Table S2). PFA100® measurements, with prolongation of closure-time after EPI induction, were also very similar in family C, symptoms and phenotype, however, differed notably between generations.

### Low Vitamin D serum levels were found in vascular type EDS patients

Serum Vitamin D level was additionally available in fourteen individuals investigated. 57 % were found to have low and critically low values (Fig. 3). One patient (ID8) was below threshold, despite current oral substitution (Additional file 2: Table S2). No patient reported any form of kidney or liver disease that might influence Vitamin D biosynthesis.

## Discussion

This is, to our best knowledge, the first study to analyze plasmatic and cellular coagulation in a unique number of 22 vascular type EDS affected individuals.

EDS reports are generally based on case scenarios and very few studies include bigger number of patients, generally from different EDS subtypes [11, 15, 16, 18, 19]. Concordantly, evidence for coagulation disorders associated with the disease comes from surgical cases and variances in the female reproduction cycle [20–22]. In 1991 *Anstey* et al. conducted a study in 51 patients of different EDS types and revealed heterogeneous abnormalities in platelet aggregation and plasmatic coagulation [16]. Here, we investigated exclusively vascular type EDS and reveal dysfunctional platelets as the most prominent disorder with a prevalence of 50 % based on single time-point blood analysis and a corresponding clinical hemorrhagic diathesis (Table 1). Especially thrombocyte stimulation by collagen, epinephrine and ADP was altered (Fig. 1/Additional file 2: Table S2). Epinephrine is a weak agonist itself, but an important catalyzer of aggregation after initial degranulation. Thus prolongation of stable aggregates might be seriously affected.

The study’s biggest limitation is the observational character based on single time-point measurements at different laboratories. Nationwide inclusion of patients and immediate sample analyses demand the participation of diverse institutes, and the use of modern, standardized test methods with clearly defined pathological ranges copes with this limitation, as the validation of results for eight patients with two functional platelet tests could demonstrate. Confounding bias in pre-analytics was tried to exclude as much as possible by evaluation of disturbing values. Additionally, analysis should be broadly available and not restricted to specialized laboratories or experimental methods.

Our findings are coherent with previously published EDS cases [16, 18, 23]. In pilot studies from the 1970s *Deliyannis* et al. and *Karaca* et al. were able to show reduced aggregation of platelets from control patients with collagen from affected patients and vice versa. Arneson et al. could restore impaired aggregation by addition of soluble fibronectin, an extracellular matrix glycoprotein, in experimental studies [24–26]. Electron microscopic examination of platelets has suggested altered distribution and function of granules [27–29]. Additionally, thrombocyte derived growth factors might influence *COL3A1* expression in non-vascular tissues [30]. Whether our results are due to dysfunctional receptor expression or impaired degranulation could not be verified in this study, yet warrants further dedicated research.

Based on the cohort size we can safely exclude other factors to noticeably influence coagulation, like thrombocyte volume, FXIII deficiency, vWS, or plasmatic coagulation, as reflected by normal Quick and PTT [15, 31–33]. Normal screening tests, however, cannot exclude mild deficiencies or functional impairment [34, 35].

Since detailed coagulation analysis is not available in emergency situations, evidence for targeted substitution must come from investigations under normal conditions. Our study provides evidence for the application of thrombocyte- and clot-formation-directed substances in case of arterial bleedings. The use of desmopressin, tranexamic acid, activated factor VII, factor VIII and platelet transfusion have been reported in EDS [15, 18, 33, 36]. Experimental data, however, only exist for desmopressin, shown to restore normal bleeding time in a total of 21 children with undefined types of EDS in two studies [15, 37]. This effect is most likely due to an increase in FVIII and vWF from endothelial reservoirs, although in-vitro experiments have also suggested a direct effect on thrombocyte granules [29, 38].

**Fig. 2:**
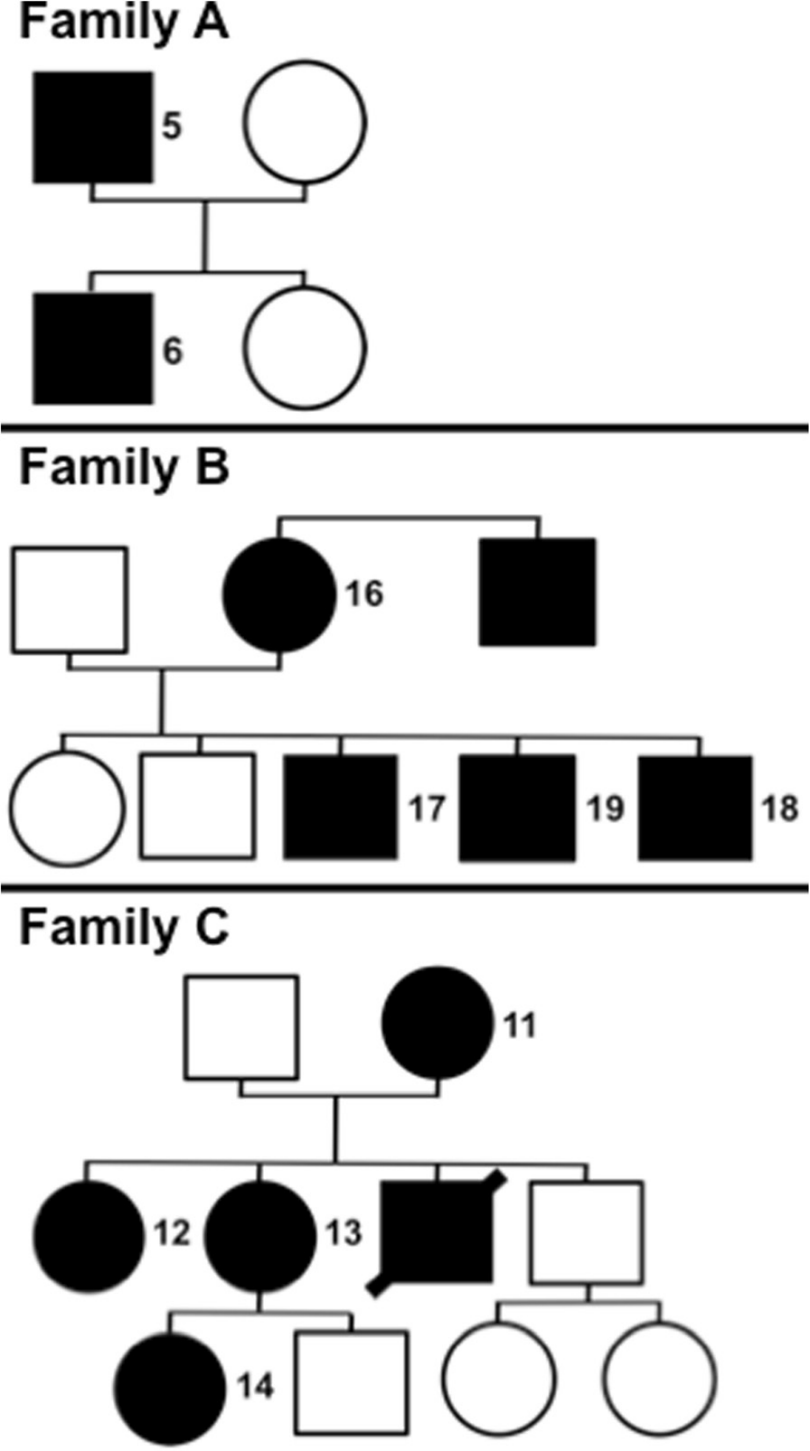
Pedigrees of enrolled families: In family A father and son are affected by vascular type EDS, daughter and mother are not. The father (ID5) was the index person. In family B the mother (ID16) was the index person. She and three of her sons were enrolled in the study. Her brother is diagnosed having a heart aneurysm but could not be enrolled in the study. In family C one daughter was the index person (ID12). She, her sister, the mother and the niece were enrolled. Her brother died on a ruptured abdominal aortic aneurysm at young age. (square = male; circle = female; filled = affected/diseased; empty = no symptoms occurred; crossed out = deceased; numbers refer to patient ID in other figures and tables; patients with number assigned on the right side indicate family members enrolled in the study)

A clear clinical type assignation in EDS is very difficult since the peculiarity of symptoms varies notably among patients and is only partly reflected by the genotype, as observed in our cohort (Table 1, Fig. 2). Variability within families has been described before, but until now not for coagulation defects [2, 3]. Our results from three different families with a total of ten members suggest, that there is no correlation of genotype, clinical symptoms and eventual coagulation disorder, are, however, limited by the small number (Fig. 2). Nevertheless a dedicated family evaluation is advisable whenever an index person is diagnosed.

**Fig. 3:**
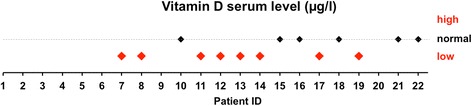
Vitamin D serum levels: The graph shows qualitative alterations in Cholecalciferole serum levels. Bold red signs indicate deviation from the normal range (dotted line). The quantitative values for each patient and test are depicted in Additional file 2: Table S2

Of special interest are the low serum levels of Vitamin D in 8 of 14 tested individuals, which has never been investigated in EDS before (Fig. 3/Additional file 2: Table S2). There is clear evidence for impaired bone mineralization and abnormal osseous structure by bone mineral density and x-ray examination from independent studies [39–41]. Furthermore, characteristic EDS-symptoms, like chronic pain, tendon weakness and myopathy, are closely related to bone integrity [42, 43]. Deficiency has been epidemiologically associated with cardiovascular disease, clear molecular mechanisms are, however, missing [44]. Diagnosis of Vitamin D is fairly cheap and oral substitution with cholecalciferol well tolerated. Thus screening of EDS patients for Vitamin D deficiency seems advisable, albeit proof of principle for oral substitution affecting the vascular phenotype has yet to be done in a bigger cohort. Additional benefit of oral substitution in vascular type EDS patients might result from anti-inflammatory and regenerative effects on chronic low-grade inflammation of the vasculature [31, 45].

## Conclusion

In a detailed functional analysis of plasmatic and cellular coagulation in a cohort of 22 patients with vascular type EDS we found a high prevalence of 50 % for thrombocyte dysfunctions. This might severely affect hemostasis in emergency bleeding situations and require targeted therapy. Elaborate coagulation screening is thus advisable in every, especially vascular type EDS patient, as well as their relatives and should include modern platelet aggregation tests. Additionally, we have analyzed Vitamin D serum levels for the first time in EDS, showing a deficiency in a respectable number of patients that might be associated with EDS characteristic symptoms.

## Abbreviations

ADP, adenosinetriphosphate; CHST14, gene name: carbohydrate sulfotransferase 14; COL, collagen; COL3A1, gene name: collagen type III alpha 1 chain; CRP, C-reactive protein; CT, closure time in PFA100® measurement; EDS, Ehers-Danlos syndrome; EIA, enzyme immunoassay; ELISA, enzyme linked immunosorbent assay; EPI, epinephrine; FVIII, Factor VIII in the coagulation cascade; FXIII, Factor XIII of the coagulation cascade; Hb, hemoglobin content; HPLC, high performance liquid chromatography; ID, identification number; PFA, platelet function analyzer; PLOD1, gene name: procollagen-Lysine,2-Oxoglutarate 5-Dioxygenase 1; PTT, activated partial thromboplastin time; vWF, von Willebrand factor; vWS, von Willebrand syndrome

## Additional files

Additional file 1: Table S1. Blood count and plasmatic coagulation laboratory results: The table shows the results for each patient, listed to patient ID according to Table 1, with unit and normal measurement range. Bold red values show deviation from the normal range. Stroked out values are not available in the specific laboratory of examination. (DOCX 74 kb) Additional file 2: Table S2. von Willebrand, Vitamin D and platelet diagnostics: The table shows the results for each patient, listed to patient ID according to Table 1, with unit and normal measurement range in brackets depending on the respective laboratory where the analysis was performed. For Vitamin D and functional platelet analysis, normal ranges differ among those depending on the commercial test used. Bold red values show deviation from the normal range. Stroked out values are not available in the specific laboratory of examination (n.a.d. = no applicable disease). (DOCX 111 kb) Additional file 3: Figure S1. von Willebrand Syndrome diagnostics: The graph shows qualitative alterations in vWF diagnosis. Bold red signs demonstrate deviation from the normal range (dotted line). (TIF 213 kb)
