# Supplementary material for: Vascular type Ehlers-Danlos syndrome is associated with platelet dysfunction and low vitamin D serum concentration
Source: Orphanet J Rare Dis. 2016 Aug 3;11:111. doi: 10.1186/s13023-016-0491-2 (PMC4971646; doi:10.1186/s13023-016-0491-2)
Supplement: Additional file 1: Table S1. — Blood count and plasmatic coagulation laboratory results: The table shows the results for each patient, listed to patient ID according to Table 1, with unit and normal measurement range. Bold red values show deviation from the normal range. Stroked out values are not available in the specific laboratory of examination. (DOCX 74 kb) [file 13023_2016_491_MOESM1_ESM.docx]

| **ID** | **Hemo-globin**  **(g/dL)**  **(11.6-16)** | **Thromb**  **count**  **(10^6^/mm^2^)**  **(150-350)** | **Thromb**  **volume**  **(fL)**  **(7.5-11.5)** | **C-react**  **protein**  **(mg/dL)**  **(**≤**0.5)** | **Quick**  **(%)**  **(70-120)** | **PTT**  **(s)**  **(25-36)** | **Fibrinogen**  **(g/L)**  **(1.6-4.0)** | **Factor XIII**  **(µg/dL)**  **(75-150)** |
| --- | --- | --- | --- | --- | --- | --- | --- | --- |
| 1 | 13.6 | 298 | - | - | 113 | 30.3 | 3.0 | - |
| 2 | 13.8 | 235 | 10.0 | 0.32 | 105 | 24.9 | 3.0 | 134 |
| 3 | 13.6 | 290 | 9.0 | 0.06 | - | 30.1 | 3.9 | **151** |
| 4 | 15.2 | 254 | - | 0.05 | 96 | 31.0 | 2.5 | 113 |
| 5 | 15.9 | 216 | - | 0.2 | 89 | 31.0 | 2.3 | 87 |
| 6 | 14.0 | 182 | - | 0.05 | 61 | 36.0 | 2.4 | 107 |
| 7 | 15.6 | 271 | 8.7 | 0.3 | 115 | 25.8 | 3.4 | 114 |
| 8 | 13.8 | **404** | **5.8** | 0.05 | 104 | 32.0 | 2.6 | 123 |
| 9 | 14.9 | 183 | 9.0 | 0.27 | 101 | 30.5 | - | - |
| 10 | **11.5** | 246 | - | 0.05 | 77 | 32.0 | 2.3 | 117 |
| 11 | 11.6 | 293 | - | 0.22 | 115 | 30.1 | 2.9 | 77 |
| 12 | 14.3 | 315 | 11.1 | 0.46 | 119 | 28.6 | 3.5 | 75 |
| 13 | 13.2 | 317 | 11.7 | 0.11 | 101 | 31.4 | 2.8 | 85 |
| 14 | 13.2 | 337 | 9.7 | 0.14 | 104 | 31.0 | 4.0 | 127 |
| 15 | 12.9 | 327 | 10.6 | 0.5 | 108 | 27.0 | 3.7 | 147 |
| 16 | 12.9 | 189 | - | 0.5 | 90 | 32.0 | 2.6 | 154 |
| 17 | 13.6 | 265 | - | 0.5 | 78 | 29.0 | 2.1 | 129 |
| 18 | 11.9 | 302 | - | 0.5 | 82 | 35.0 | 2.8 | 110 |
| 19 | 13.2 | 245 | - | 0.5 | 88 | **40.0** | 2.7 | 139 |
| 20 | 14.4 | 242 | 11.8 | - | 106 | 26.5 | 3.0 | 85 |
| 21 | 13.1 | 337 | 8.2 | - | 93 | 26.6 | **1.6** | 120 |
| 22 | 13.3 | 324 | - | 0.05 | 101 | 33.0 | 2.7 | 99 |
