# Supplementary material for: Vascular type Ehlers-Danlos syndrome is associated with platelet dysfunction and low vitamin D serum concentration
Source: Orphanet J Rare Dis. 2016 Aug 3;11:111. doi: 10.1186/s13023-016-0491-2 (PMC4971646; doi:10.1186/s13023-016-0491-2)
Supplement: Additional file 2: Table S2. — von Willebrand, Vitamin D and platelet diagnostics: The table shows the results for each patient, listed to patient ID according to Table 1, with unit and normal measurement range in brackets depending on the respective laboratory where the analysis was performed. For Vitamin D and functional platelet analysis, normal ranges differ among those depending on the commercial test used. Bold red values show deviation from the normal range. Stroked out values are not available in the specific laboratory of examination (n.a.d. = no applicable disease). (DOCX 111 kb) [file 13023_2016_491_MOESM2_ESM.docx]

| **ID** | **vWF activity**  **(%)** | **vWF**  **antigen**  **(IU/dL)** | **Vitamin D3**  **(µg/L)** | **PFA100^©^**  **(s)** | | **Born**  **Aggregation**  **(%)** | | | |
| --- | --- | --- | --- | --- | --- | --- | --- | --- | --- |
| 1 | - | - | - | - | | - | | | |
| 2 | 132 (50-180) | >599 (highly elevated rheumatoid factor) | - | EPI 100 (84-160) | ADP 67 (68-121) | n.a.d. | | | |
| 3 | **194** (61-179) | **189** (unknown) | - | **EPI 60** (84-160) | - | **EPI 30** (60-90) | ADP 61 (60-90) | **Col 95** (60-80) | - |
| 4 | - | - | - | EPI 126 (82-150) | *-* | - | ADP 79 (>60) | **Col 32** (>60) | Rist 79 (>60) |
|  |  |  |  |  |  | ***ASS responder*** | | | |
| 5 | - | - | - | EPI 124 (82-150) | *-* | - | ADP 90 (>60) | Col 83 (>60) | Rist 91 (>60) |
| 6 | - | - | - | EPI 126 (82-150) | *-* | - | **ADP 37** (>60) | **Col 5** (>60) | **Rist 58** (>60) |
| 7 | 159 (46-173) | 166 (42-176) | **4.3** (30-120) | - | | EPI 78 (60-90) | ADP 77 (60-90) | Col 91 (60-90) | - |
| 8 | **46** (47-173) | **54** (60-200) | **22.0** (20-40)  **substitution** | **EPI 199** (84-160) | ADP 108 (68-121) | - | ADP 90 (>60) | Col 87 (>60) | Rist 88 (>60) |
| 9 | - | - | - | **EPI 219** (84-160) | ADP 92 (68-121) | **EPI >40µmol/l** | ADP normal | Col normal | Rist normal |
| 10 | 136 (61-179) | 138 (70-180) | 135 (50-75) | **EPI 164** (84-160) | ADP 79 (68-121) | n.a.d. | | | |
| 11 | - | - | **26** (30-120) | - | | **EPI 91** (60-90) | ADP 80 (60-90) | Col 86 (60-90) | - |
| 12 | - | - | **10** (30-120) | - | | **EPI 104** (60-90) | **ADP 103** (60-90) | **Col 100** (60-90) | - |
| 13 | - | - | **6.5** (30-120) | - | | **EPI 91** (60-90) | ADP 77 (60-90) | Col 87 (60-90) | - |
| 14 | - | - | **7.6** (30-120) | - | | **EPI 97** (60-90) | ADP 89 (60-90) | **Col 100** (60-90) | - |
| 15 | **227** (61-179) | **197** (70-180) | 122 (50-75) | EPI 96 (84-160) | ADP 65 (68-121) | n.a.d. | | | |
| 16 | 64 (46-146) | 62 (50-130) | 107 (50-75) | **EPI 207** (84-160) | ADP 121 (68-121) | - | | | |
| 17 | **39** (46-146) | 54 (50-130) | **40.0** (50-75) | **EPI 271** (84-160) | **ADP 158** (68-121) | - | | | |
| 18 | 101 (46-146) | 92 (50-130) | 55.0 (50-75) | **EPI 246** (84-160) | ADP 101 (68-121) | - | | | |
| 19 | 95 (46-146) | 95 (50-130) | **42.0** (50-75) | **EPI 232** (84-160) | ADP 99 (68-121) | - | | | |
| 20 | 128 (61-179) | 134 (57-174) | - | **EPI 192** (84-160) | **ADP 163** (68-121) | EPI 70 (60-90) | **ADP 45** (60-90) | **Col 99** (60-80) | **Rist 57** (60-80) |
| 21 | - | 160 (48-173) | n.a.d. | **EPI 300** (84-160) | ADP 95 (68-121) | - | | | |
| 22 | 70 (60-130) | 74 (50-150) | 29.3 (20-70) | **EPI 227s** (<190) | **ADP 111** (<110) | - | | | |
