# Supplementary figures and images for: Vascular type Ehlers-Danlos syndrome is associated with platelet dysfunction and low vitamin D serum concentration
Source: Orphanet J Rare Dis. 2016 Aug 3;11:111. doi: 10.1186/s13023-016-0491-2 (PMC4971646; doi:10.1186/s13023-016-0491-2)

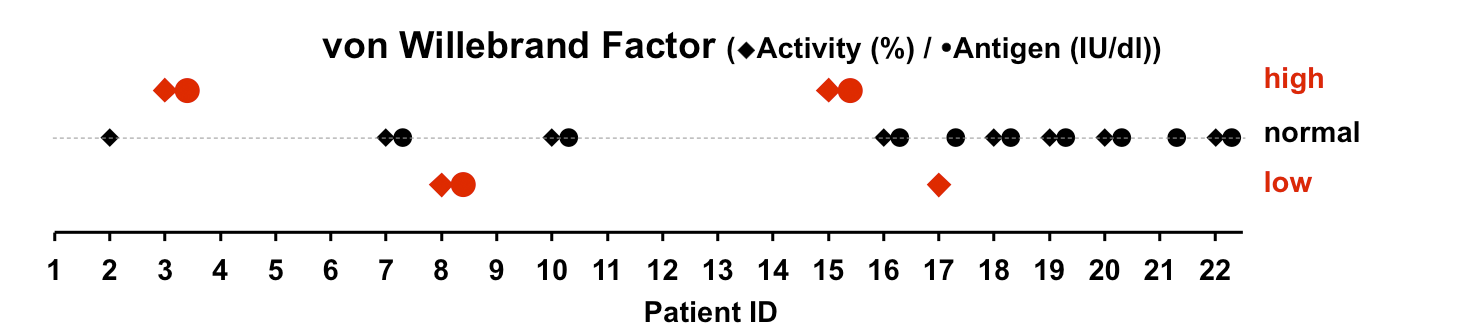

Supplement: Additional file 3: Figure S1. — von Willebrand Syndrome diagnostics: The graph shows qualitative alterations in vWF diagnosis. Bold red signs demonstrate deviation from the normal range (dotted line). (TIF 213 kb) [file 13023_2016_491_MOESM3_ESM.tif]
